# Tackling worms in children: school programmes can work – for eyes too

**Published:** 2013

**Authors:** David Addiss

**Affiliations:** Director: Children Without Worms, Taskforce for Global Health, Decatur, Georgia, USA.

**Figure:**
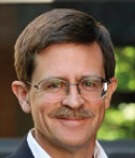
David Addiss

An estimated 800-900 million children worldwide are affected by intestinal worms. The majority of these live in Asia and Africa.

There are three main types of intestinal worms:

- roundworms *(Ascaris)*
- whipworm *(Trichuris)*
- hookworm (two species: *N. americanus* and *A. duodenale)*

These worms – also known as soil-transmitted helminths (STH) – primarily affect children and women of childbearing age. Infected children may become anaemic and tired, and have difficulty learning. The worms absorb vital nutrients from the intestine, which can affect children's growth, health, and nutrition. Some studies have shown that children with high levels of infection have impaired cognitive (brain) function, which may be reversed by treatment to get rid of the worms.^1^ Pregnant women infected with hookworm also may suffer from anaemia, which leads to low birth weight and affects the baby's chances of survival and future health.

## Transmission

Intestinal worms are usually associated with poor sanitation, which includes poor access to toilets (latrines) and hand washing facilities. When there is no access to toilets and people defecate in the fields, the soil becomes contaminated. Worm eggs can persist for months or even years in the soil, given the right conditions. Roundworm and whipworm are transmitted when soiled hands contaminated with human faeces come into contact with the mouth (faecal-oral contact). Hookworm larvae penetrate the skin of the feet that come into contact with contaminated soil.

## Preventing transmission

The use of well-maintained latrines and hand washing after using the toilet and before eating food are the major health education messages.

## Tackling worms

The World Health Organization (WHO) recommends preventive chemotherapy- known as mass drug administration (MDA) – when the prevalence of worm infection in children is above 20% (i.e. more than 20 out of every 100 children have worms). MDA provides children with a worm-free interval during which they can absorb nutrients from their food and grow. The treatment is repeated every 6-12 months, depending on the intensity of infection in the community. In areas of heavy transmission, it is necessary to treat repeatedly for several years.

School children are often heavily infected with worms and, because schools are easily accessible, treatment and prevention of intestinal worm infections have become part of school health programmes in the countries where this is a problem. One day in every 6-12 months is usually designated as de-worming day and treatment is given together with hygiene and sanitation lessons. The teachers usually hand out the de-worming drugs and ensure the children take them correctly.

At a global level, the de-worming programme has been slow to reach everyone, but this has started to change. Johnson & Johnson and GlaxoSmithKline now give sufficient free drugs to reach all school-age children in the 112 countries where children are at risk of infection. At present, 45 countries are receiving the donated medicines. In addition, the United Nations Children's Fund (UNICEF) and other organisations provide treatment to pre-school children as part of vaccination or vitamin A supplementation programmes. As a global community, we are now organising and rallying around a common vision for the control of intestinal worms.

Even though de-worming drugs are now available, free of charge, organisational and technical capacity are required to get the drugs to the schools and have them administered effectively. Countries are now preparing their plans of action, which includes not only treatment for intestinal worms (soil-transmitted helminths) but also other neglected tropical diseases. Once the plans have been developed, countries can request drugs for the control of worms through WHO. They need to demonstrate that they have the infrastructure and funding in place to deliver the drugs to rural as well as urban schools, to train teachers to administer the medicines to the children, and to mobilise communities to participate.

There are two major priorities if we are to eliminate worms by 2020:

1. **Scaling up.** This is particularly important in the larger countries (India, Nigeria, Ethiopia, Indonesia, and Democratic Republic of Congo) so that all the children who need the medication have access to it.
2. **Sustainability.** As well as increasing MDA coverage, there must be an emphasis on hygiene education. This can be achieved through collaboration with the water, sanitation, and hygiene (WASH)sector. Thich is critical for prevention of transmission and for long-term success (see page 27).

## What can eye care workers do?

Being aware that these school-based de-worming programmes exist and that they provide a health focus for the schools can be helpful. De-worming day can be an excellent platform for other public health interventions, such as health education, orvision screening. Children in school will benefit most from an integrated health package-not just de-worming, not just eye testing -to enable them to live a more healthy life.

**Figure:**
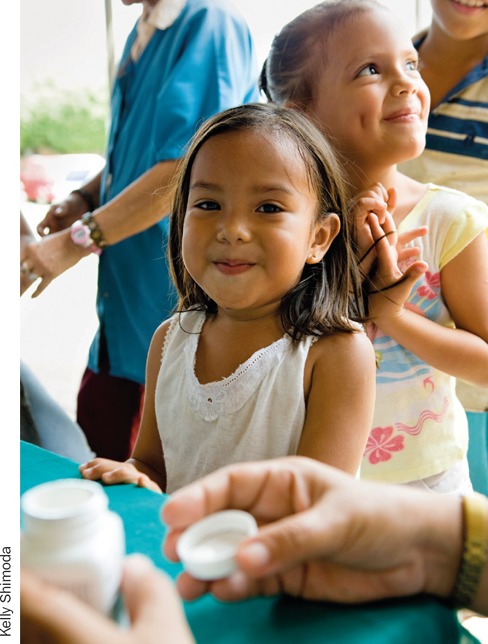
Children line up to receive their deworming medicine. NICARAGUA

## References

[1] Nokes C Grantham-McGregor SM Sawyer AW Cooper ES, et al. (1992) Moderate to heavy infections of Trichuris trichiura affect cognitive function in Jamaican school children. Parasitology 1992;104:539-547 1641252 10.1017/s0031182000063800

