# Supplementary material for: Tackling worms in children: school programmes can work – for eyes too
Source: Community Eye Health. 2013;26(82):29–31. (PMC3756647)
Supplement: Related texts

# Brief report: At a glance: the core neglected tropical diseases (NTDs)

**Published:** 2013

**Table:** 

Trachoma	Onchocerciasis	Soil-transmitted	Lymphatic filariasis	Schistosomiasis
					
	Trachomatous trichiasis	A woman blinded by onchocerciasis	Adult female *Ascaris lumbricoides* worm	Elephantiasis due to lymphatic filariasis	Dipstick testing to detect haematuria. The sample on the left is negative for haematuria -the other two are both positive
**Where**	Africa Latin America Yemen China India Australia South-East Asia Pacific Islands(see **www.trachomaatlas.org)**	Africa Latin America Yemen	Worldwide(see **www.thiswormyworld.org)**	Africa, Asia Latin America Pacific Islands(see **www.thiswormyworld.org)**	Africa Asia Latin America(see **www.thiswormyworld.org**)
**How**	Discharge from infected eyes spreads via fingers, fomites and eye-seeking flies (especially *Musca sorbens)*	Acquired bythebiteof an infected blackfly *(Simulium sp.)*	Eggs are passed out in faeces and then swallowed by another host *{Ascaris, Trichuris)* or develop into infective larvae and penetrate intact skin (hookworm)	Acquired bythebiteof infected mosquitoes	Acquired by contact with standing fresh water (e.g. lakes) in which there are infected snails
**Who**	Pre-school-age children have the highest prevalence of active trachoma. The prevalence of blindness from trachoma increases with age	People living near rivers where blackflies breed	People living in communities with poor access to water and sanitation People who walk barefoot are at risk of hookworm infection	Children aquire the infection, but sequelae tend to occur in adults	Children and adults who play, work or undertake chores on the edge of lakes
**What**	Inflammation of the conjunctiva (active trachoma) Conjunctival scarring Trichiasis Corneal opacification	Nodules under the skin Pruritus Thickening of skin or loss of skin pigment Punctate keratitis Sclerosing keratitis Uveitis Chorioretinitis Optic atrophy	Often asymptomatic May contribute to anaemia leading to fatigue and lethargy	Often asymptomatic Enlargement of the limbs, genitals or breasts, with secondary skin changes	**Acute infection:**fever, itchy rash **Chronic infection:**often asymptomatic; haematuria, renal failure, bladder cancer (urinary schistosomiasis); liver failure (intestinal schistosomiasis)
**Test**	Clinical diagnosis	Skin snip Slitlamp examination of eye	Stool microscopy for eggs	Examination of night blood smear for microfilariae	Urine/stool microscopy for eggs
**Treat**	**Individual:** Tetracycline eye ointment twice daily for 6 weeks, or single doseazithromycin, 20mg/kg (maximum lg), for active trachoma Surgery for trichiasis
**Individual:** Ivermectin 150μg/kg once a month for 3 months	**Individual:** Single dose albendazole 400mg	**Individual:** Albendazole 400mg for 21 days Washing of swollen parts to reduce risk of secondary infection and progression of elephantiasis	**Individual:** Two doses of praziquantel 20mg/kg taken 6 hours apart after food
	**Population:** SAFE strategy	**Population:** Mass distribution of ivermectin	**Population:** Mass distribution of albendazole or mebendazole. Improved access to safe methodsfordisposal of human faeces	**Population:** Mass distribution of DEC+albendazole, or (ifonchocerciasis co-endemic) ivermectin+albendazole	**Population:** Mass distribution of praziquantel Improved access to safe methods for disposal of human faeces Education
